# 非抑制型离子色谱法同时测定矿泉水中硼酸和偏硅酸

**DOI:** 10.3724/SP.J.1123.2023.09025

**Published:** 2023-12-08

**Authors:** Zhanqiang YANG, Fangfang ZHANG, Chunxia HAN, Hongguo ZHENG

**Affiliations:** 1.赛默飞世尔科技(中国)有限公司,上海201203; 1. Thermo Fisher Scientific (China) Co., Ltd., Shanghai 201203, China; 2.赛默飞世尔科技(中国)有限公司,四川成都610023; 2. Thermo Fisher Scientific (China) Co., Ltd., Chengdu 610023, China

**Keywords:** 非抑制型离子色谱, 硼酸, 偏硅酸, 矿泉水, non-suppressed ion chromatography, boric acid, silicic acid, mineral water

## Abstract

建立了利用非抑制电导检测法同时测定矿泉水中硼酸及偏硅酸(以SiO_3_^2-^计)含量的方法。在以Dionex IonPac^TM^ AS20作为分析柱、流速为1.0 mL/min、色谱柱温度为30 ℃、以6 mmol/L氢氧化钠溶液和60 mmol/L甘露醇作为流动相、进样体积50 μL的条件下,偏硅酸和硼酸在8 min内实现有效分离,SiO_3_^2-^和硼酸分别在0.25~100 mg/L和0.5~100 mg/L范围内线性关系良好(相关系数均为0.9999)。SiO_3_^2-^的方法检出限(MDL)和方法定量限(MQL)分别为0.078 mg/L和0.26 mg/L,硼酸的方法检出限(MDL)和方法定量限(MQL)分别为0.18 mg/L和0.60 mg/L。以实际样品为基质在不同添加水平下进行加标试验,SiO_3_^2-^和硼酸的平均加标回收率为97.3%~105.3%,相对标准偏差(RSD)<0.9% (*n*=6),满足检测要求。该方法前处理简单,样品过0.22 μm水系滤膜后直接进样分析。在优化的分析条件下分别对9种市售矿泉水中硼酸和偏硅酸含量进行了检测,9种市售矿泉水均未检出硼酸,6种检测出偏硅酸,含量为18.70~62.08 mg/L,均与厂家包装上所标注的浓度范围一致。该方法可用于饮用矿泉水、实验室用水等领域中,同时也为半导体行业用超纯水中硼酸和偏硅酸的同时检测提供了参考。

## Abstract (en)

Boron and silicon are widely distributed in nature; in water, these compounds typically present in the forms of boric acid and silicic acid, respectively. The maximum allowable levels of silicic acid and boric acid in water are stipulated in relevant national and industry standards, such as GB 8538-2022. Quality changes in water, which are of great significance in water-quality evaluations, can be understood in terms of its silicic acid and boric acid contents. Boric acid content is usually determined by ion exclusion chromatography, whereas silicic acid content is usually determined by postcolumn derivatization. Therefore, traditional methods cannot achieve the simultaneous determination of silicic acid and boric acid contents in water. Modern ion chromatography has been widely used in the detection of ionic compounds, such as anions, cations, organic acids, organic amines, amino acids, and sugars. Boric (p*K*_a_=9.24) and silicic (p*K*_a_=9.77) acids are weak acids that dissociate into ionic states under alkaline conditions. Although these compounds cannot be tested using suppressed ion chromatography, they can be retained on ion chromatography columns. In this study, a method based on nonsuppressed conductance detection was established for the simultaneous determination of boric acid and silicic acid in water. The contents of boric acid and silicic acid were detected by nonsuppressed ion chromatography using a Dionex IonPac^TM^ AS20 analytical column. The chromatographic conditions were as follows: flow rate, 1.0 mL/min; column temperature, 30 ℃; eluent, 6 mmol/L sodium hydroxide solution and 60 mmol/L mannitol; and sample injection volume, 50 μL. The effective separation of silicic acid and boric acid was achieved within 8 min. SiO_3_^2-^ and boric acid demonstrated good linear relationships in the concentration ranges of 0.25-100 and 0.5-100 mg/L (correlation coefficients, 0.9999), respectively. The method detection (MDL) and quantification (MQL) limits were 0.078 and 0.26 mg/L for SiO_3_^2-^, and the MDL and MQL limits were 0.18 and 0.60 mg/L for boric acid. The average recoveries of boric acid and SiO_3_^2-^ (*n*=6) were 97.3%-105.3%. Moreover, the relative standard deviations were less than 0.9% for boric acid at four spiked levels and less than 0.30% for SiO_3_^2-^ at three spiked levels. Thus, the method meets detection requirements. The pretreatment method is very simple, and the sample can be directly injected through a 0.22 μm water filtration membrane and into the column. The boric acid and silicic acid contents in nine mineral drinking water samples were determined under the optimized analytical conditions. Boric acid was not detected in these nine samples, but silicic acid was detected in six samples. The silicic acid contents detected were between 18.70 and 62.08 mg/L, which was consistent with the concentration ranges marked on the manufacturers’ packaging. The proposed method can be used for the determination of boric acid and silicic acid in mineral drinking water and laboratory water, and provides a reference for the simultaneous detection of boric acid and silicic acid in ultrapure water used in the semiconductor industry.

自从Small等^[1]^首次提出现代离子色谱概念以来,离子色谱已被广泛应用于阴、阳离子等离子型化合物的检测^[2⇓-4]^。目前抑制电导检测模式已逐渐成为离子色谱领域的主要发展趋势^[5⇓-7]^。但抑制型离子色谱法并不适用于p*K*_a_>7的弱酸。因为在抑制模式中,常用的氢氧根淋洗液经过抑制后的产物为水,会将待测物周围环境从碱性变成中性,从而导致p*K*_a_>7的弱酸电离程度极大的减弱,不利于电导检测器的测定。因此针对p*K*_a_>7的弱酸常用其他方式进行检测,如非抑制电导法^[8,9]^和柱后衍生紫外检测法^[10]^等。

硼在自然界中主要以硼酸和硼酸盐的形式存在。最新的《生活饮用水卫生标准》(GB 5749-2022)规定硼的含量需小于1.0 mg/L;许多国家和组织也制定了相应的标准,如欧盟规定饮用水中硼的限值为1.0 mg/L,新西兰饮用水标准中硼的限值为1.4 mg/L。因此,随着硼工业的发展及人们对水中硼污染重视的提高,水中硼含量的检测越来越引起人们的广泛关注。现有已发表的文献中关于矿泉水中硼酸含量测定的方法主要有甲亚胺-H或姜黄素分光光度法^[11]^、电感耦合等离子体原子发射光谱法^[12,13]^、离子色谱法^[14]^等。甲亚胺-H分光光度法和姜黄素分光光度法均需要对硼酸进行衍生化操作,操作步骤繁琐,尤其是样品量比较大时,这两种方法耗时较多。现行标准DB51/T 1698-2013中推荐使用离子排斥色谱法测定样品中的硼酸,该方法能够有效减小强电离离子化合物对硼酸测定的干扰,但选择性相对较差,小分子有机酸与硼酸出峰相近甚至共洗脱,从而易对硼酸的定性定量造成干扰。

硅是人体所必需的微量元素,一般以偏硅酸的形式存在于水中,对人体有益。GB 8537-2018 《食品安全国家标准 饮用天然矿泉水》将偏硅酸列为矿泉水七项界限指标之一。传统的硅酸盐检测方法主要有氢氟酸转化分光光度法、重量法、气体分割连续流动光度分析^[15,16]^等,而国标GB 8538-2022中的方法则是采用硅钼黄分光光度法和硅钼蓝分光光度法检测偏硅酸。由于这些方法存在操作繁琐、消耗试剂量大、污染环境等缺点,新的分析方法不断地被建立。

离子色谱是分离阴、阳离子的首选方案,不少学者尝试考虑用离子色谱法来测定样品中的硅酸盐^[8⇓-10,17⇓⇓⇓-21]^。薛京昌等^[18]^分别采用抑制型-紫外柱后衍生法、非抑制型-紫外柱后衍生法测定矿泉水中的硅酸盐含量,紫外柱后衍生法在不同程度上存在操作复杂、费时费力等问题。刘肖等^[19]^利用AS11-HC作为分析柱,淋洗液发生器在线产生KOH梯度淋洗测定矿泉水中的硅酸盐,该报道仅针对单一硅酸盐的检测。Dasgupta等^[20]^采用双电导检测器串联的方式实现对常规阴离子和弱有机酸的同时检测,其第一个电导检测器为标准的抑制型电导检测系统,在两个电导检测器之间增加一个自制的交换单元从而实现在进入第二个电导检测器之前对流动相加碱的功能。在碱性条件下,有机弱酸的解离程度增加,信号增强。但由于第二个检测器前死体积较大,易导致色谱峰扩散,有机酸转换效率较低,信号增强程度有限,同时也缺少商品化的交换单元,因此该方法未得到普及。

通常测定硼酸采用离子排斥色谱法,测定硅酸根采用柱后衍生紫外法。因此传统方法不能实现同时对水质中硅酸根和硼酸含量的测定。考虑到硼酸(p*K*_a_=9. 24)和偏硅酸(p*K*_a_=9. 77)均为极弱酸,在碱性条件下有一定解离,虽不能用抑制型离子色谱法测试,但在离子色谱柱上仍有保留。故本文采用非抑制型离子交换色谱法,通过优化色谱柱和淋洗液条件,实现硼酸和偏硅酸的同时检测分离。

## 1 实验部分

### 1.1 仪器与试剂

Dionex^TM^ Inuvion Core离子色谱仪、Dionex^TM^ AS-DV自动进样器、Dionex^TM^ IC Pure^TM^水纯化系统、Fisher Scientific^TM^11203超声波清洗器、Thermo Scientific^TM^ Chromeleon^TM^色谱分析数据系统(CDS)软件均为赛默飞世尔科技有限公司产品。

九水合硅酸钠(Na_2_SiO_3_·9H_2_O,纯度98%)、硼酸(H_3_BO_3_,纯度99.5%,)、甘露醇(C_6_H_14_O_6_, 纯度98%)均购自Merck公司;50%氢氧化钠水溶液(赛默飞世尔科技有限公司);实验所用其他试剂均为分析纯;实验用水均为去离子水(18.2 MΩ·cm)。

### 1.2 离子色谱条件

色谱柱:Dionex IonPac^TM^AG20保护柱(50 mm×4 mm)+Dionex IonPac^TM^AS20色谱柱(250 mm×4 mm);淋洗液:6 mmol/L氢氧化钠溶液+60 mmol/L甘露醇;流速:1.0 mL/min;进样体积:50 μL;柱温:30 ℃。

### 1.3 标准溶液与淋洗液的配制

准确称取0.375 g Na_2_SiO_3_·9H_2_O,溶于80 mL水中,并定容至100 mL,制得1000 mg/L的SiO_3_^2-^标准溶液。

准确称取0.1 g H_3_BO_3_,溶于80 mL水中,并定容至100 mL,制得1000 mg/L的H_3_BO_3_标准溶液。

称取10.9 g甘露醇置于1000 mL容量瓶中,加水溶解;加入0.31 mL 50%氢氧化钠溶液,充分混匀溶解后加水稀释至刻度线,制得淋洗液,备用。

### 1.4 实际样品的前处理

样品过0.22 μm水系滤膜后直接进样分析。

## 2 结果与讨论

### 2.1 色谱柱及流动相选择

在离子色谱系统中淋洗液的组成及其浓度对目标化合物的保留影响重大。用于阴离子分析的流动相主要包括氢氧化物、碳酸盐-碳酸氢盐体系等,考虑到碳酸盐-碳酸氢盐的洗脱能力较强,硼酸和偏硅酸较难保留。另一方面,硼酸和偏硅酸的p*K*_a_值与碳酸-碳酸氢盐体系的缓冲pH范围较为接近,可能会对其电离产生影响。故综合以上两点,本研究采用氢氧根体系作为淋洗液。

首先考察了Dionex IonPac^TM^AS20、Dionex IonPac^TM^AS11-HC、Dionex IonPac^TM^AS19色谱柱的分离效果。Dionex IonPac^TM^AS20色谱柱亲水性较好,柱容量较大,采用较低浓度淋洗液即可将偏硅酸和硼酸与常规离子实现分离,且分离度良好(见图1)。在非抑制模式中,背景电导通常与淋洗液浓度成正比,低浓度淋洗液则具有较低的基线背景,对待测物来讲可获得更低的检出限。Dionex IonPac^TM^AS11-HC柱容量虽然与Dionex IonPac^TM^AS20相差不大,但Dionex IonPac^TM^AS11-HC柱亲水性相对较差,主要用于常规阴离子及有机酸分析。Dionex IonPac^TM^AS19柱容量适中,主要用于卤氧化物、卤代乙酸的分析,虽然硼酸和偏硅酸也可实现分离,但分离度相对较小。故综合考虑,本研究采用Dionex IonPac^TM^AS20作为实验分析柱。在该非抑制模式下,信号峰为倒峰。为方便对比和定量数据获取,通过极性切换方式将倒峰转化为正峰。

**图1:**
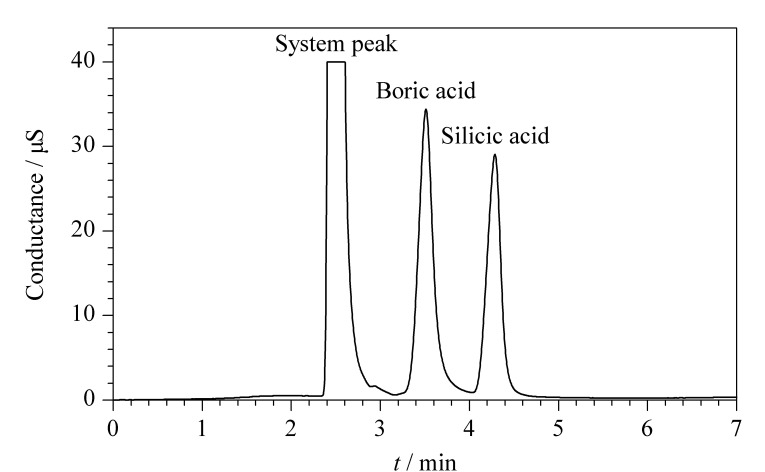
IonPac^TM^ AS20色谱柱分离硼酸和偏硅酸的色谱图

根据文献[22]报道,硼酸可以与多羟基化合物进行络合,从而形成比硼酸酸性更强的络合酸,从而有利于硼酸的测定。因此本实验在氢氧化钠流动相的基础上分别考察了不同浓度的甘露醇对实验结果的影响,见图2。实验结果表明,当甘露醇浓度为0 mmol/L时,硼酸在偏硅酸后边洗脱,峰扩散严重,半峰宽较大,不利于定性定量分析;当添加30 mmol/L甘露醇时,硼酸与流动相中甘露醇进行络合配位,形成更加稳定的阴离子,表现为硼酸在偏硅酸前面出峰,但其色谱峰形前伸,存在肩峰现象,其原因推测为硼酸与甘露醇形成配位比不同的混合物;而当进一步增大甘露醇浓度至60 mmol/L时,硼酸和偏硅酸保留时间基本变化不大,但硼酸的色谱峰峰形得到较好的改善;继续增加甘露醇的浓度,硼酸色谱峰基本不变。故最终确定流动相中甘露醇的浓度为60 mmol/L。

**图2:**
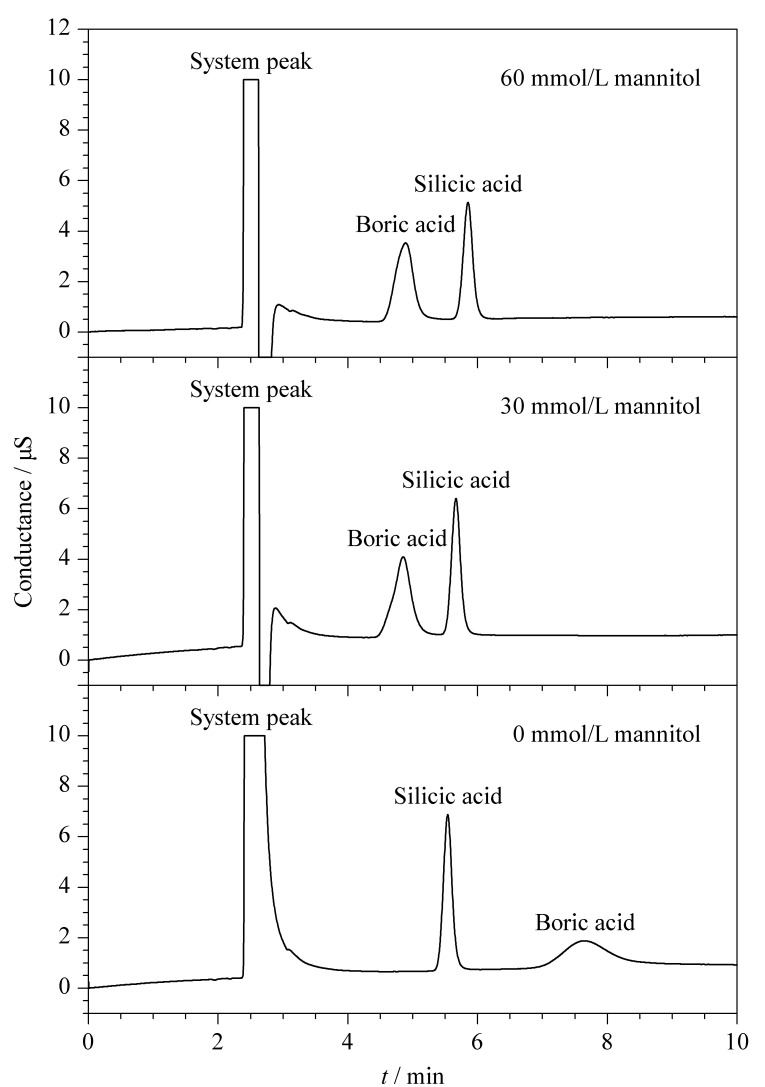
流动相中甘露醇浓度对硼酸和偏硅酸分离的影响

### 2.2 方法学考察

#### 2.2.1 标准曲线及重复性

用超纯水配制不同浓度的SiO_3_^2-^和硼酸系列标准工作溶液,在选定的色谱条件下进样分析,以目标物的峰面积(*y*, μS·min)为纵坐标,标准溶液质量浓度(*x*, mg/L)为横坐标,建立标准曲线。硼酸和SiO_3_^2-^的线性范围、线性方程和相关系数(*R*)见表1。可以看出,SiO_3_^2-^和硼酸在选定的浓度范围内均具有良好的线性,*R*为 0.9999。

**表1:** 硼酸和SiO_3_^2-^的线性范围、回归方程、相关系数

Analyte	Linear range/(mg/L)	Regression equation	*R*
Boric acid	0.5-100	*y*=0.0845*x*+0.0215	0.9999
Silicate	0.25-100	*y*=0.074*x*+0.0032	0.9999

*y*: peak area, μS·min; *x*: mass concentration of anions, mg/L.

重复性是判断分析方法是否适用的重要指标之一。本文选取10 mg/L的硼酸和SiO_3_^2-^连续进样9次,硼酸和SiO_3_^2-^保留时间的相对标准偏差(RSD)≤0.09%,峰面积RSD≤0.21%,进一步说明仪器方法具有良好的稳定性,具体重复性结果见表2。

**表2:** 硼酸和SiO_3_^2-^定性、定量的重复性(*n*=9)

Analyte	Retention time				Peak area		
	Average/ min	RSD/%			Average/ (μS·min)		RSD/%
Boric acid	5.280	0.09		0.8921		0.21	
Silicate	6.367	0.07		0.7611		0.20	

#### 2.2.2 检出限和定量限

分别以信噪比(*S/N*)等于3和10所对应的目标物浓度作为仪器检出限和仪器定量限^[3]^。当样品水直接进样分析,仪器检出限和仪器定量限即为水中SiO_3_^2-^和硼酸的方法检出限和定量限(见表3)。

**表3:** 硼酸和SiO_3_^2-^的仪器检出限、仪器定量限、方法检出限和方法定量限

Anion	IDL/ (mg/L)	IQL/ (mg/L)	MDL/ (mg/L)	MQL/ (mg/L)
Boric acid	0.18	0.60	0.18	0.60
Silicate	0.078	0.26	0.078	0.26

#### 2.2.3 加标回收率和精密度

本方法选择市售矿泉水作为本底样品,分别对SiO_3_^2-^和硼酸进行不同浓度等级的低中高三水平加标测试;考虑到样品中硼酸未检出,故对硼酸又进行了定量限水平的加标试验。结果表明,SiO_3_^2-^和硼酸的平均回收率均为97.3%~105.3%,证明该方法的准确度较高;检测结果的RSD均在0.9%以内,证明该方法稳定性好。详细结果见表4所示。

**表4:** 矿泉水样品中硼酸和SiO_3_^2-^在不同添加水平下的回收率和相对标准偏差(*n*=6)

Analyte	Background/ (mg/L)	Spiked/ (mg/L)	Recovery/ %	RSD/ %
Boric acid	ND	0.6	97.3	0.89
		10	104.2	0.29
		20	105.3	0.17
		30	104.2	0.14
Silicate	18.2	10	100.5	0.30
		20	102.7	0.25
		30	101.9	0.08

ND: not detected.

#### 2.2.4 实际样品测定

在优化的实验条件下,选取9种市售饮用矿泉水作为分析样品进行实验,不同的饮用矿泉水中偏硅酸含量相差较大,但均未检测到硼酸的存在,具体实验结果见表5,检出偏硅酸的饮用矿泉水中偏硅酸的含量均与其商标中标示值范围一致。

**表5:** 矿泉水样品中偏硅酸和硼酸的测定结果

Sample No.	Determination results/(mg/L)	
	Silicic acid^*^	Boric acid
1	58.34	ND
2	62.08	ND
3	49.93	ND
4	ND	ND
5	37.67	ND
6	20.08	ND
7	ND	ND
8	18.70	ND
9	ND	ND

* Silicic acid contents were calculated with SiO_3_^2-^ contents. ND: not detected.

## 3 结论

建立了一种利用非抑制型离子色谱同时测定水中硼酸和偏硅酸含量的方法。与传统检测方法相比,本方法通过一针进样即可获得水中硼酸和偏硅酸的含量。该方法前处理简单,无需柱后衍生,减少了工作量,降低了成本。此外,目前较为通用的纯水标准中也都明确规定了偏硅酸、硼酸的含量,尤其是电子级超纯水中,对偏硅酸和硼酸的要求更为苛刻。鉴于其含量相对较低,故本方法应用在超纯水中还需进一步优化条件。
